# Using Malaise Traps and Metabarcoding for Biodiversity Assessment in Vineyards: Effects of Weather and Trapping Effort

**DOI:** 10.3390/insects13060507

**Published:** 2022-05-27

**Authors:** Marvin Kaczmarek, Martin H. Entling, Christoph Hoffmann

**Affiliations:** 1 Julius Kühn Institute—Federal Research Institute for Cultivated Plants, Institute for Plant Protection in Fruit Crops and Viticulture, Geilweilerhof, D-76833 Siebeldingen, Germany; christoph.hoffmann@julius-kuehn.de; 2 Institute for Environmental Sciences—iES Landau, University of Koblenz-Landau, Fortstraße 7, D-76829 Landau in der Pfalz, Germany; entling@uni-landau.de

**Keywords:** metabarcoding, Malaise trap, insect monitoring, viticulture, insect diversity, taxa richness, biomass, species accumulation, trap exposure, sampling method

## Abstract

**Simple Summary:**

A strong decline in insect biomass and biodiversity has been observed in the past decades. Long-term monitoring programs are important to understand the reasons for changes in species occurrence, which are mostly attributed to habitat destruction, intensified agriculture, invasive organisms, and climate change. Metabarcoding, a procedure for identifying insect species in bulk samples based on their DNA sequences, provides a method to replace otherwise time-consuming species identification in a time- and cost-efficient way. In this study, we examined how weather and trapping effort affect biomass and biodiversity of Malaise trap catches in vineyards using metabarcoding. Most insects were caught during warm and hot weather. We observed that, as the number of trapping days and sites increased, there was a very high accumulation of taxa due to species with low abundance. The results can help in developing monitoring programs. Common species can be extensively surveyed with less effort, whereas rare taxa require excessive effort to be completely surveyed due to a lack of saturation. Thus, metabarcoding can play an important role in conducting monitoring by offsetting the additional effort required to detect rare species by making identification less time consuming and costly

**Abstract:**

Metabarcoding is a powerful tool for ecological studies and monitoring that might provide a solution to the time-consuming taxonomic identification of the vast diversity of insects. Here, we assess how ambient weather conditions during Malaise trap exposure and the effort of trapping affect biomass and taxa richness in vineyards. Biomass varied by more than twofold with weather conditions. It increased with warmer and drier weather but was not significantly related with wind or precipitation. Taxa richness showed a saturating relationship with increasing trapping duration and was influenced by environmental and seasonal effects. Taxa accumulation was high, increasing fourfold from three days of monthly trap exposure compared to continuous trapping and nearly sixfold from sampling at a single site compared to 32 sites. The limited saturation was mainly due to a large number of singletons, such as rare species, in the metabarcoding dataset. Metabarcoding can be key for long-term insect monitoring. We conclude that single traps operated for up to ten days per month are suitable to monitor the presence of common species. However, more intensive trapping is necessary for a good representation of rare species in biodiversity monitoring. The data collected here can potentially guide the design of monitoring studies.

## 1. Introduction

Terrestrial insects have strongly declined during recent decades, with important consequences for the functioning of the world’s ecosystems [1,2,3,4,5]. A significant loss of biodiversity and biomass is reported, the reasons for which are attributed to habitat destruction, intensified agriculture, invasive organisms, and climate change [4]. To be able to record the further course of these trends and the reasons responsible for them, systematic monitoring of terrestrial insects, as it is performed in only a few monitoring programs [6,7,8,9], is essential. However, in addition to the long-term influences of, e.g., agriculture and climate change, environmental and methodological conditions during sampling could have a direct effect on the insect diversity collected and should thus be considered when evaluating data from ecological surveys [10].

Insect activity depends on the season and ambient weather conditions [11,12,13]. While warm, dry weather can promote activity, especially in the summer months, it can be reduced in cold and rainy weather [8,13,14]. Most flying insects are trapped at hot and sunny conditions after it has rained, although there are differences among taxa [12,15]. Nevertheless, short-term weather conditions only affect activity during the event. Long-term changing temperature or precipitation patterns due to climate change, however, have a lasting impact on insect populations [16]. For example, flight activity can decrease at above-average temperatures in summer [8,14].

Long-term insect monitoring can quantify trends in biomass, species richness, species composition, and species abundance and allow conclusions about the reasons for changes by including environmental parameters [17]. Because of the vast diversity of insects, the large number of trapped individuals, and the thus time-consuming and costly identification of species, long-term studies, especially when based on morphological species identification, usually either depend on adequate funding and high effort or are limited to some of these proxies or focus on indicator groups [6,18,19]. Additionally, reducing the monthly effort, i.e., shortening the sampling period, may be useful from both a conservation and economic perspective to reduce environmental impacts and costs [17]. In any case, the selection of the trapping method already turns the focus on certain species groups [10].

Arthropods can be trapped with a large variety of trap types, including pitfall traps, suction traps, window traps, pan traps, bait traps, light traps, and Malaise traps [20,21]. Malaise traps are a widely used trap type in biodiversity surveys and monitoring because they are easy to handle and capture a huge variety of flying insects and also wingless arthropods, with Diptera and Hymenoptera being by far the most-collected taxa [10,19,22]. The large quantity of insects caught in Malaise traps, however, makes it laborious to process bulk samples [6]. Time-intensive species identification often relies on the few available experts, a problem known as the taxonomic impediment [23]. Metabarcoding can be a solution to the challenge of the high time required for taxonomic identification by identifying taxa in a time- and cost-efficient way [24].

Using high-throughput sequencing, metabarcoding combines DNA sequences in the region of the cytochrome c oxidase I (COI) gene of similar specimens into operational taxonomic units (OTUs) [25]. OTUs can be assigned to barcode index numbers (BINs) by comparison with reference sequences in the Barcode of Life Data System (BOLD) [25]. BINs allow a taxonomic assignment based on reference sequences. The proportion of OTUs that can be assigned to BINs or species depends on the coverage of species in the databases. Even though not all BINs are assigned to Linnean names, they still often correspond well to the species level [24]. Thus, BINs can be a good proxy for species diversity to derive trends also for arthropod groups that are not well covered in the BOLD library [26]. If BINs have an assignment to species, the comparison with red lists also allows short-term conclusions about the occurrence of endangered or invasive species [27]. A disadvantage of metabarcoding is that, in contrast to morphological species identification and counting of individuals, no quantified species abundance is recorded [28]. Thus, no accurate conclusion can be made about the abundance of individual species in single samples [29]. For replicate samples, the relative abundance can be derived from the frequency of species occurrence [26,30]. In addition, there are promising approaches to estimate the relative abundance of a species based on the reads of DNA sequences in a sample [31,32,33].

In this study, we used Malaise traps and metabarcoding to collect and identify insects in vineyards in southwest Germany. As part of a larger effort to establish an insect monitoring program for viticulture, our first aim was to assess if biomass in southwest German viticulture is affected in a comparable way by environmental conditions as it has already been demonstrated in other ecosystems [8,11,12,13,14,15]. We tested the following hypothesis: (H1) (a) cool temperatures, (b) precipitation, and (c) wind reduce the biomass of trapped insects. The question if vineyards are saturated or unsaturated ecosystems is tested in the two further hypotheses: (H2) (a) taxa richness and (b) cumulative taxa richness show a saturating relationship with trapping duration; and (H3) a larger number of trapping sites increase cumulative taxa richness.

## 2. Materials and Methods

### 2.1. Study Area

**Figure 1:**
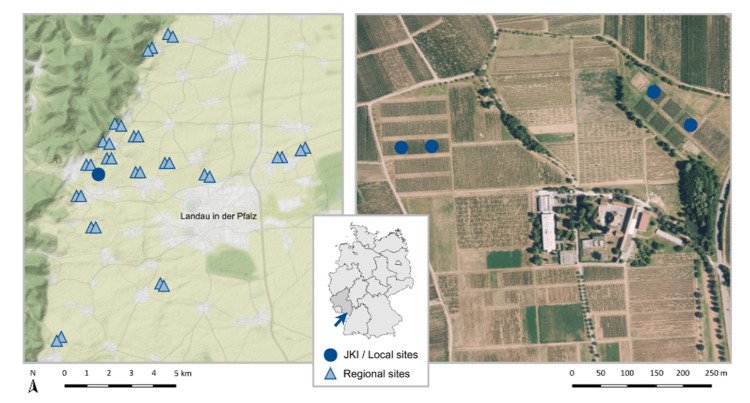
Study area with the location of the Julius Kühn institute (JKI) and the 32 regional sampling sites (**left**, map data by OpenStreetMap, under ODbL) and the institutes area with the location of the four local sampling sites (**right**, image data by © GeoBasis-DE/LVermGeoRP (2022)). The arrow indicates the study area in Rhineland-Palatinate on the map of Germany.

Our study area is located in the German wine-growing region Palatinate (Figure 1), which has a warm temperate climate with warm summers, an average annual temperature of 11.1 °C, and a total annual precipitation of 677.7 mm [34,35]. We sampled locally in the vineyards of the Julius Kühn Institute (JKI) in Siebeldingen (49.218350° N, 8.045650° E, Rhineland-Palatinate, Germany) and regionally in 32 vineyards in the surrounding area (49.273280° N, 8.020602° E/49.147516° N, 8.175736° E, Rhineland-Palatinate, Germany).

### 2.2. Sampling

We conducted the local sampling in four vineyards in the institute area. In each vineyard, we installed one Malaise trap (standard SLAM trap, MegaView Science Co., Ltd., Taichung, Taiwan) from 4 June to 2 October 2021. We filled collecting bottles with 300 mL ethanol denatured with about 1% methyl ethyl ketone (EtOH MEK) and changed them at least every five days to preserve already-trapped material. Ethanol was subsequently replaced in all samples. The sampling period was divided into four 30-day cycles with four trapping intervals each. Each cycle, we collected insect material on the 3rd, 8th, 16th, and 30th day, resulting in intervals of 3, 5, 8, and 14 trapping days per month, respectively. For each of the four vineyards, we pooled the material from the four 30-day cycles for each duration of trapping, resulting in four bulk samples with a total trapping duration of 12, 20, 32, and 56 trapping days, respectively.

We conducted the regional sampling in 32 vineyards and sampled two years from April to September in 2020 and 2021. Each month, we installed one malaise trap (first three months Malayse traps with a combination of black and white net, ENTO SPHINX s.r.o., Pardubice, Czech, from then on standard SLAM traps) for three consecutive days in each vineyard, resulting in a total of 36 trapping days per site. Collecting bottles were filled with 300 mL of 70% EtOH MEK, and collected material was stored in undiluted EtOH MEK. We pooled the material for each site of each year, resulting in two bulk samples per vineyard and 64 samples in total.

### 2.3. Environmental Conditions

We retrieved daily environmental data for temperature, radiation, precipitation, air humidity, and wind speed from a weather station, which is located in the institute area [35]. We then calculated the mean of the variables for each trapping interval of the local sampling using the daily mean for temperature, air humidity, and wind speed and the daily total for radiation and precipitation (Table 1).

### 2.4. Biomass

For the local sampling, we weighed the wet biomass material of each trap for each interval after placing it in a sieve and letting the liquid drip off (Table S1, Supplementary Materials). Liquid at the bottom of the sieve was additionally dapped on a paper tissue.

### 2.5. Taxa Richness

DNA metabarcoding and bioinformatics (using VSEARCH v.2.9.1 [36], Cutadapt v.1.18 [37], and Geneious v.10.2.5 (Biomatters, Auckland, New Zealand)) of the 16 bulk samples of the local sampling and the 64 bulk samples of the regional sampling were conducted by AIM (Advanced Identification Methods GmbH) following the methods of Hausmann et al. [19] and Morinière et al. [38] (Supplementary Methods, Supplementary Materials), with species identification based on high-throughput sequencing (HTS) data grouped to genetic clusters (OTUs), blasted, and assigned to BINs and species. We filtered the results table for OTUs with a Hit-%-ID value in BOLD ≥ 97% and an assignment to a BIN and condensed BINs that occurred more than once into one entry. We then filtered the results table for BINs with an assignment to a species and condensed species that occurred more than once into one entry. For the regional sampling, we condensed BIN lists of the two years for each of the 32 vineyards. The numbers of BINs were used as a value for taxa richness (Tables S2 and S3, Supplementary Materials). According to their occurrence in the four vineyards of the local sampling and the 32 vineyards of the regional sampling, respectively, BINs were classified into subsets with taxa with high (caught at more than three-quarters of the sites), medium (caught at more than one and up to three-quarters of the sites), and low (caught at up to one-quarter of the sites).

### 2.6. Data Analysis

All analyses were conducted using R v.4.0.4 (R Foundation for Statistical Computing, Vienna, Austria) [39] and RStudio v.1.2.5033 (RStudio, Inc., Boston, MA, USA) [40] with the R packages *car* [41] for performing linear regressions and *ggplot2* [42] and *vegan* [43] for creating figures. Additionally, we used Inkscape v.1.0.2-2 (Inkscape Team) for creating figures [44]. We explored the data for distribution patterns. We investigated the effect of the environmental variables on the daily biomass and the effect of the trapping duration on the taxa richness and the accumulated taxa richness, including the presence subsets, by performing linear regression analyses with type III ANOVA using a significance level of *p* < 0.05. Due to correlations between temperature, radiation, and air humidity as well as precipitation and wind speed, we used separated models for each environmental variable (Figure S1, Supplementary Materials).

## 3. Results

### 3.1. Biomass

**Figure 2:**
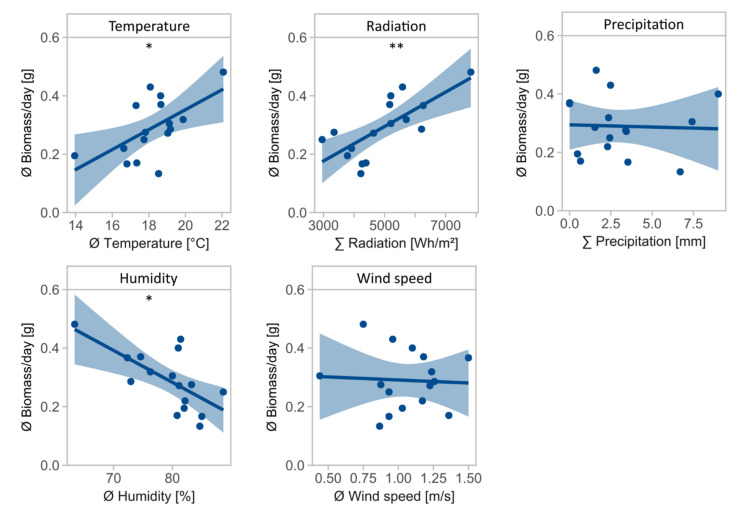
Mean daily biomass in g for environmental variables temperature in °C, radiation in Wh/m^2^, precipitation in mm, air humidity in %, and wind speed in m/s. Asterisks indicate significant effects of environmental variables on daily biomass (significance codes: ** *p* < 0.01, * *p* < 0.05).

Daily biomass was influenced by temperature (*F* = 7.5, Df = 1, *p* = 0.016, Table 2), radiation (*F* = 15.8, Df = 1, *p* = 0.001), and air humidity (*F* = 11.1, Df = 1, *p* = 0.005), with temperature and radiation positively associated and air humidity negatively associated with daily biomass (Figure 2). Precipitation (*F* = 0.0, Df = 1, *p* = 0.884) and wind speed (*F* = 0.0, Df = 1, *p* = 0.846) had no significant effect on daily biomass.

### 3.2. Taxa Richness

**Figure 3:**
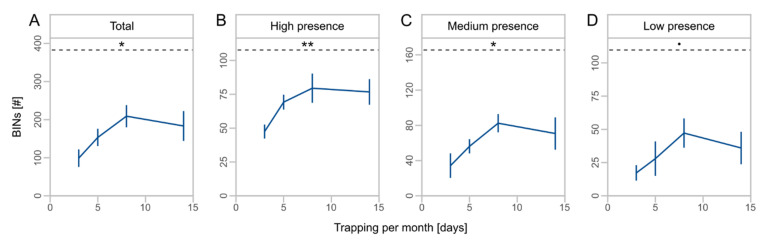
Mean number of barcode index numbers (BINs) ± SD for different days of monthly trapping for total BINs (**A**) and subsets (**B**–**D**) with high (taxa caught at 4 sites), medium (taxa caught at 2 or 3 sites), and low presence (taxa caught at 1 site). Asterisks indicate significant effects of monthly trapping duration on number of BINs (significance codes: ** *p* < 0.01, * *p* < 0.05, • *p* < 0.1). Dashed lines indicate the mean total number of BINs. Note different scale of y-axes.

We obtained a total of 1494 OTUs from metabarcoding of the local sampling, which were assigned to 836 BINs (Table 3). BINs were assigned to 18 orders, 157 families, and 461 species (Table S4, Supplementary Materials). The orders Diptera (43.2%), Hymenoptera (14.5%), Coleoptera (13.9%), Lepidoptera (11.5%), and Hemiptera (10.6%) accounted for the largest proportions of BINs. The duration of monthly trapping affected the number of total BINs (*F* = 7.2, Df = 1, *p* = 0.018, Table 2) and taxa with high (*F* = 9.5, Df = 1, *p* = 0.008) and medium presence (*F* = 7.2, Df = 1, *p* = 0.018). For low presence taxa, we observed no significant effect of the monthly trapping duration (*F* = 3.7, Df = 1, *p* = 0.075). Taxa richness increased by twofold from three to eight days of monthly trapping with no further increase to 14 days (Figure 3A), and higher proportions of total BINs were captured for high presence taxa compared to medium and low presence taxa (Figure 3B–D).

**Figure 4:**
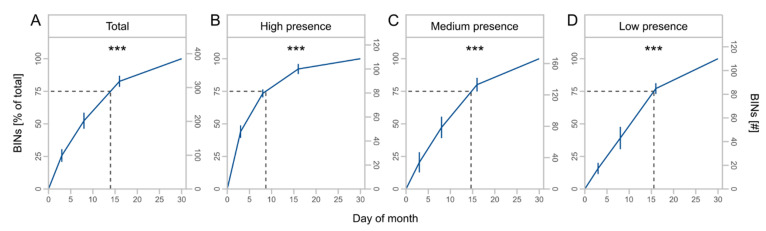
Mean cumulative proportion and number of BINs ± SD at different days of monthly trapping for total BINs (**A**) and presence subsets (**B**–**D**) with high (taxa caught at 4 sites), medium (taxa caught at 2 or 3 sites), and low presence (taxa caught at 1 site). Asterisks indicate significant effects of monthly trapping duration on number of BINs (significance code: *** *p* < 0.001). Dashed lines indicate the day of month at 75% of the total BINs.

The cumulative number of BINs (*F* = 71.4, Df = 1, *p* < 0.001) and taxa with high (*F* = 43.6, Df = 1, *p* < 0.001), medium (*F* = 85.1, Df = 1, *p* < 0.001), and low presence (*F* = 47.7, Df = 1, *p* < 0.001) increased with increasing duration of trapping (Table 2). The number of BINs was nearly four times greater at 30 days of monthly trapping than at 3 days (Figure 4A) but differed for subsets with decreasing saturation from high to low presence taxa (Figure 4B–D).

**Figure 5:**
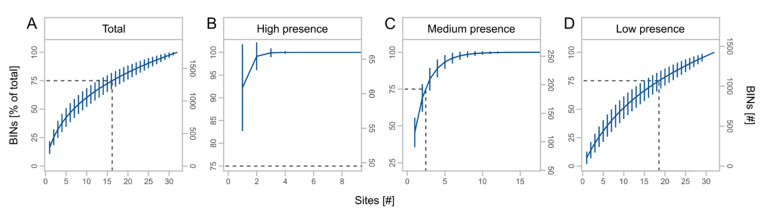
Mean cumulative proportion and number of BINs ± SD at different number of sites for total BINs (**A**) and subsets (**B**–**D**) with high (taxa caught at 25 to 32 sites), medium (taxa caught at 9 to 24 sites), and low presence (taxa caught at 1 to 8 sites). Dashed lines indicate the number of sites at 75% of the total BINs. Note different scale of x- and y-axes.

We obtained a total of 3245 OTUs from the metabarcoding of the regional sampling, which were assigned to 1748 BINs (Table 3). BINs were assigned to 19 orders, 227 families, and 1020 species, with the orders Diptera (38.0%), Hymenoptera (21.9%), Coleoptera (15.0%), Lepidoptera (11.3%), and Hemiptera (8.8%) accounting for the largest proportions of BINs (Table S5, Supplementary Materials). The cumulative number of BINs increased with the number of sites, but with a slight saturation effect (Figure 5A). BIN numbers for high presence taxa reached saturation at about three traps (Figure 5B) and for medium presence taxa at about eight traps (Figure 5C). For rare species, we observed almost no saturation effect (Figure 5D). In total, 75% of BINs were recorded using at least 17 traps. For high, medium, and low presence taxa, 75% of BINs were recorded using at least one, three, and 19 traps, respectively.

## 4. Discussion

Biomass increased with higher temperature and radiation levels, which corresponds to our first hypothesis (H1a), where we expected biomass to decrease with cool temperatures. Temperature and radiation are strongly coupled, in particular during the summer period [45,46]. Both were observed to positively influence insect activity rates in earlier studies [1,8,11], with ambient temperature having a direct effect on body temperature and thus flight ability [47]. However, other studies reported that at above-average temperatures in the summer months, the linear relationship breaks down and activity decreases [8,14], so that a temperature optimum curve could actually have been expected. We did not observe such a decrease at high temperatures, presumably because temperatures in the study year of the local sampling were rather moderate compared to the three preceding years [35].

In contrast to the second part of our first hypothesis but similar to Welti et al. [8], biomass did not decrease with precipitation (H1 b). Although rain events reduce flight activity in various insects [14], the effect of short-term weather conditions becomes less apparent when considering multi-day trapping intervals [15]. However, biomass decreased with increasing air humidity. While we have found a negative effect of air humidity on biomass, other studies have found both positive and negative effects for different groups or species of insects [12,48]. Air humidity, however, was not at extreme levels during data collection, which can cause a clear reduction in catches [12]. In addition, air humidity correlates with temperature and radiation, so that the decrease of catches with increasing high air humidity in our study may be a consequence of reduced temperature or radiation at high air humidity rather than a direct effect of air humidity itself.

Contrary to what we hypothesized, wind speed was not affecting biomass (H1 c). Mean wind speed was generally low in the present study, with daily means not exceeding 2 m/s throughout data collection of the local sampling and not affecting flight activity as it did in other studies [12]. The daily maximum wind speed reached values of more than 10 m/s during sampling [35]. As with rain, however, we expect stronger wind to be a short-term event and therefore less apparent at multi-day trapping intervals.

Almost 60% of the OTUs could be assigned to BINs, and more than 30% could be assigned to species. The distribution of BINs among orders is comparable to that of other studies with malaise traps [10]. Despite several projects to record German insects in the DNA barcode libraries [26,49,50,51,52,53,54,55,56,57,58,59,60,61,62,63,64,65], these results underline that there still is a need for further sequencing work to provide more comprehensive databases to match OTUs to BINs and species and by that to increase the informative value of monitoring programs and insect surveillance. Noticeably, only about half of the BINs of Hymenoptera and Diptera could be assigned to a species, while the proportion is higher for other insect orders such as Lepidoptera and Coleoptera. In addition to species not yet recorded in DNA barcode libraries, this can also be attributed to a higher proportion of undescribed species and “dark taxa” in these hyperdiverse orders [7,26,66]. Despite the current lack of species linked to reference sequences, the use of metabarcoding in long-term monitoring offers an advantage herein, as archived raw sequence data can be quickly reprocessed with updated databases. Today’s undescribed species, “dark taxa” without scientific names in the databases, or species whose taxonomic classification will change can be included in future analyses.

We expected taxa richness to increase with increasing trapping duration (H2 a), which was only the case up to a certain value. After a duration of eight days, there was no further increase of BINs. The lack of increase can be explained in part by a saturating effect of more common species that are caught in all trapping intervals. In addition, the environmental and seasonal conditions presumably reduced flight activity, especially in the 14-day interval compared to the 8-day interval, as the average temperature was much lower in three of the four 30-day cycles (Figure S2, Supplementary Materials). Seasonal conditions generally changed toward the end of data collection, and a decline in activity of many species toward fall and winter likely reduced the taxa richness in the later trapping intervals [13], with trapping of rare species appearing to be more affected here.

Consistent with the second part of our second hypothesis (H2 b), taxa accumulation was high with increasing monthly trapping duration. While a clear saturating effect can be seen for more common taxa, this effect increasingly weakens to a barely flattening accumulation curve for low presence taxa. As a result, complete saturation is not evident in the total number of BINs either, as it has similarly been reported in previous studies [7,67,68]. Common species are likely caught with short trapping durations, so a flattening of the accumulation curve was expected. However, some species are generally less likely to be caught, such as rare and transient or non-flying species, and are thus infrequently captured in traps [67]. In addition, Malaise traps mainly catch actively flying insects, mainly from the orders Diptera and Hymenoptera [10]. The flight period of some species can be less than one month [69], which reduces the likelihood of catching these species during short trapping periods. Yet, the non-flattening accumulation curve for rare taxa shows that even with continuous trapping duration, not all of these species can be captured.

Cumulative taxa richness increased with a greater number of sampled sites, as expected in our last hypothesis (H3), but without a clear saturation effect on the total number of BINs. As with local sampling, rare species cannot be comprehensively detected even with excessive sampling effort, whereas for more common species sampling at three to ten sites can be sufficient to record regionwide-distributed species. However, due to the higher beta diversity in the region [70], the more common species accounted for only a small proportion of the total species, and species classified as low presence taxa may be common at one site but infrequently distributed across the landscape. Comprehensively detecting species classified as those with a higher presence at local scale can hence require a higher sampling effort at regional scale.

## 5. Conclusions

We showed how the sampling effort is affecting biomass and the recorded biodiversity through weather conditions, trapping duration, and sampled sites. Biomass is the highest on warm and dry days, which increased taxa richness within Malaise trap samples. More than three-quarters of species at a site can be detected by trapping for half a month, and sampling at only three to ten sites can be sufficient to capture regionwide-distributed species. While common species in vineyards can be extensively surveyed with less effort, a complete survey of rare taxa requires high effort due to low saturation. Metabarcoding can provide comprehensive species lists and thus be an answer to the problem of time-consuming morphological identification, especially for long-term monitoring where archived data can be reprocessed with updated DNA barcode libraries. Samples from Malaise traps, however, consist of a large extent of single taxa that can be, e.g., transient or low abundant species. As recommended by Steinke et al. [67], research on the origin of these singletons should be considered in future biodiversity surveys. Here, long-term monitoring could contribute to a better understanding by providing long-term data on the recurrent abundance of single taxa at a sampling site.

## Figures and Tables

**Table 1:** Mean of daily meteorological conditions at trapping intervals during the experiment. Minimal, maximal, mean value, and SD for the environmental variables.

Variable	Unit	Min	Max	Mean	SD
Temperature	Degrees Celsius (°C)	13.9	22.1	18.2	1.7
Radiation	Watt hours per square meter (Wh/m^2^)	2956	7822	4918	1240
Precipitation	Millimeters (mm)	0.0	9.0	2.9	2.6
Air humidity	Percent (%)	63.4	88.7	79.3	6.2
Wind speed	Meters per second (m/s)	0.4	1.5	1.1	0.3

**Table 2:** Results table of linear regression analyses with type III ANOVA for the effect of the environmental variables temperature, radiation, precipitation, air humidity, and wind speed on daily biomass and for the effect of monthly trapping duration on taxa richness and accumulated taxa richness with *F*-value, degrees of freedom (Df), and *p*-value. The effects on taxa richness and accumulated taxa richness are also given for subsets of high (taxa caught at 4 sites), medium (taxa caught at 2 or 3 sites), and low presence (taxa caught at 1 site). Bold letters indicate significant effects.

	Dependent Variable	Explanatory Variable	*F-*Value	Df	*p-*Value
Environment	Biomass/day	**Temperature**	7.5	1	**0.016**
	Biomass/day	**Radiation**	15.8	1	**0.001**
	Biomass/day	Precipitation	0.0	1	0.884
	Biomass/day	**Air humidity**	11.1	1	**0.005**
	Biomass/day	Wind speed	0.0	1	0.846
Taxa richness	BINs	**Trapping duration**	7.2	1	**0.018**
	BINs (High presence)	**Trapping duration**	9.5	1	**0.008**
	BINs (Medium presence)	**Trapping duration**	7.2	1	**0.018**
	BINs (Low presence)	Trapping duration	3.7	1	0.075
Accumulated richness	BINs	**Trapping duration**	71.4	1	**<0.001**
	BINs (High presence)	**Trapping duration**	43.6	1	**<0.001**
	BINs (Medium presence)	**Trapping duration**	85.1	1	**<0.001**
	BINs (Low presence)	**Trapping duration**	47.7	1	**<0.001**

**Table 3:** Number of barcode index numbers (BINs) assigned to the 1494 operational taxonomic units (OTUs) of the local and the 3245 OTUs of the regional sampling and number of families and species assigned to BINs for the most common orders. Proportion of total in % is given in brackets.

Order	Local Sampling			Regional Sampling		
	BINs	Families	Species	BINs	Families	Species
Diptera	361 (43.2)	43 (27.4)	165 (35.8)	664 (38.0)	51 (22.5)	321 (31.5)
Hymenoptera	121 (14.5)	19 (12.1)	60 (13.0)	383 (21.9)	37 (16.3)	201 (19.7)
Coleoptera	116 (13.9)	25 (15.9)	94 (20.4)	262 (15.0)	36 (15.9)	211 (20.7)
Lepidoptera	96 (11.5)	24 (15.3)	68 (14.8)	198 (11.3)	41 (18.1)	143 (14.0)
Hemiptera	89 (10.6)	17 (10.8)	45 (9.8)	154 (8.8)	24 (10.6)	96 (9.4)
Others	53 (6.3)	29 (18.5)	29 (6.3)	87 (5.0)	38 (16.7)	48 (4.7)
Total	836	157	461	1748	227	1020

## Data Availability

Data are included in the article or Supplementary Materials.

## Supplementary Materials

The following supporting information can be downloaded at: https://www.mdpi.com/article/10.3390/insects13060507/s1, Figure S1: Correlation matrix for environmental variables temperature, radiation, precipitation, air humidity, and wind speed; Figure S2: Course of mean daily biomass and the environmental variables; Table S1: Biomass of the local sampling; Table S2: Taxa richness of the local sampling; Table S3: Taxa richness of the regional sampling; Supplementary Methods; Supplementary References; Table S4: BINs, taxonomy, and presence of the local sampling; Table S5: BINs, taxonomy, and presence of the regional sampling.
