# Supplementary material for: Using Malaise Traps and Metabarcoding for Biodiversity Assessment in Vineyards: Effects of Weather and Trapping Effort
Source: Insects. 2022 May 27;13(6):507. doi: 10.3390/insects13060507 (PMC9224819; doi:10.3390/insects13060507)
Supplement: Supplementary file 1 [file insects-13-00507-s001.zip › insects-1690677 supplementary/insects-1690677 supplementary .pdf]

Supplementary Materials for

Using Malaise traps and metabarcoding for biodiversity assessment: effects of weather and trapping effort

Marvin Kaczmarek*, Martin H. Entling and Christoph Hoffmann

* Correspondence: marvin.kaczmarek@julius-kuehn.de

Supplementary Information:

Supplementary Figures S1-S2
Supplementary Tables S1-S3
Supplementary Methods
Supplementary References

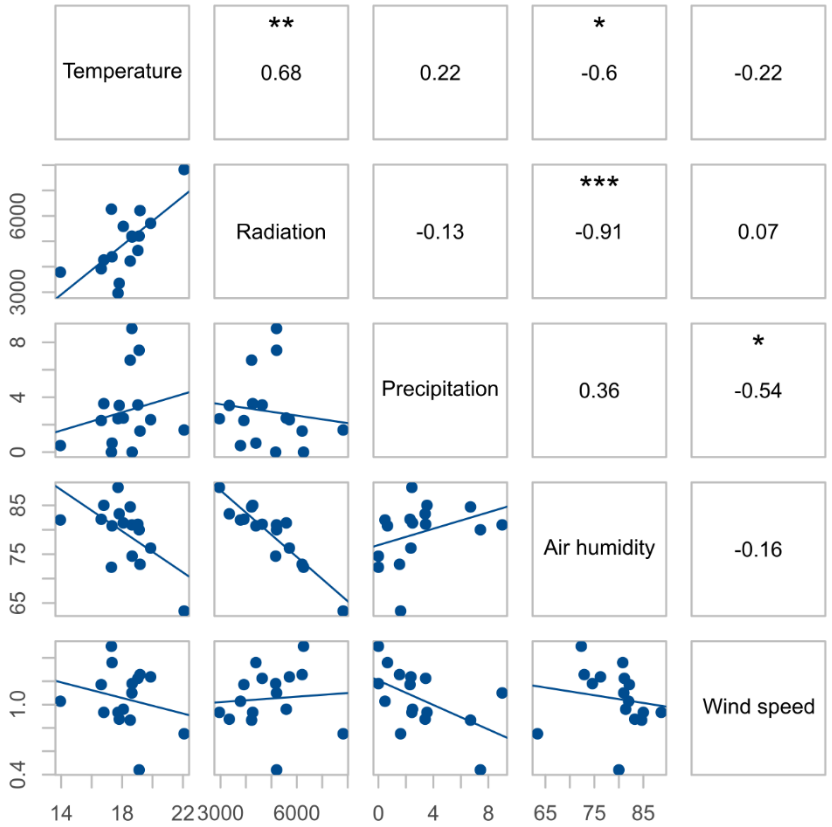

Figure S1. Correlation matrix with R-value for environmental variables temperature, radiation, precipitation, air humidity, and wind speed. Asterisks indicate significant correlation between variables based on P-values (significance codes: *** $p < 0.001$, ** $p < 0.01$, * $p < 0.05$).

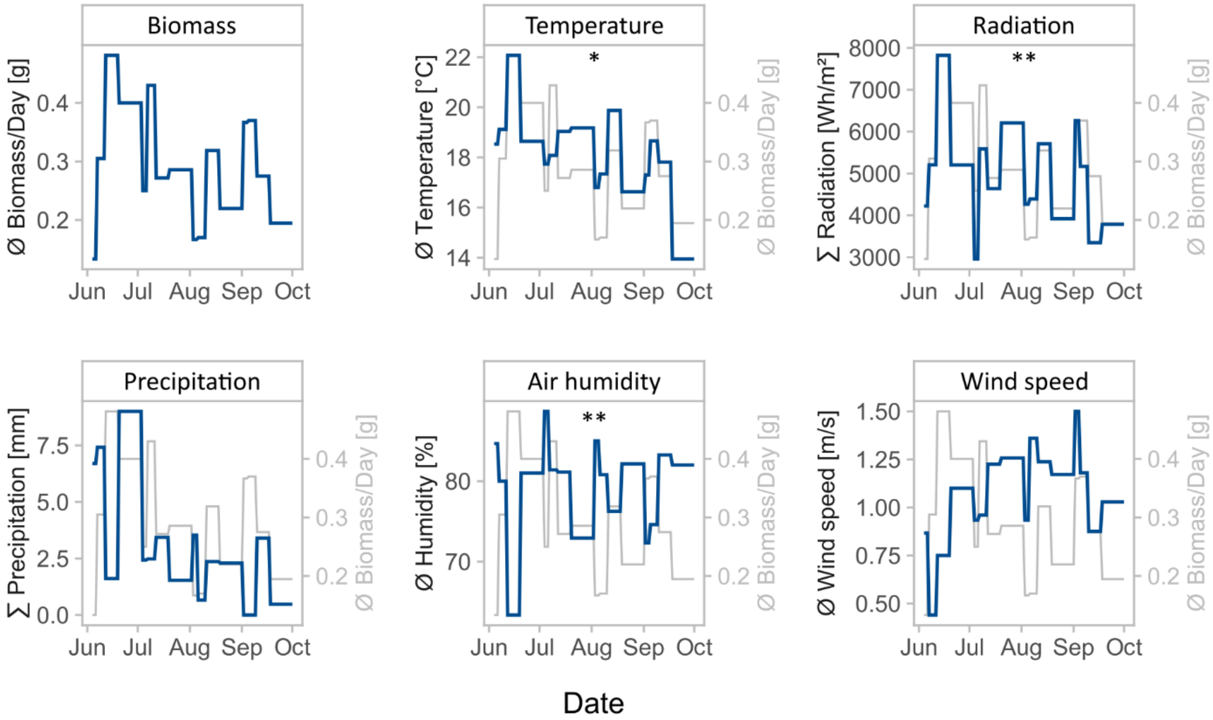

Figure S2. Course of mean daily biomass in g and the environmental variables temperature in °C, radiation in Wh/m², precipitation in mm, air humidity in %, and wind speed in m/s for each trapping interval throughout data collection. Asterisks indicate significant effects of environmental variables on daily biomass (significance codes: ** $p < 0.01$, * $p < 0.05$).

Table S1. Biomass for trap A-D of the local sampling and the mean environmental variables temperature in °C, radiation in Wh/m², precipitation in mm, air humidity in %, and wind speed in m/s for each trapping interval.

			Biomass										
Cycle	Interval	Days of trapping	A	B	C	D	Mean	Mean/Day	Temperature	Radiation	Precipitation	Air humidity	Wind
1	i1	3	0,4	0,6	0,3	0,3	0,4	0,1	18,53	4223	6,70	84,67	0,87
1	i2	5	1,3	1,6	1,1	2,1	1,5	0,3	19,12	5206	7,42	80,00	0,44
1	i3	8	2,3	5,3	3,3	4,5	3,9	0,5	22,08	7822	1,61	63,38	0,75
1	i4	14	2,8	8,4	5,4	5,8	5,6	0,4	18,64	5203	9,01	81,00	1,10
2	i1	3	0,2	1	0,8	1	0,8	0,3	17,73	2956	2,43	88,67	0,93
2	i2	5	1,2	3	2,7	1,7	2,2	0,4	18,08	5586	2,48	81,40	0,96
2	i3	8	2,3	1,1	2,5	2,8	2,2	0,3	19,04	4638	3,44	81,13	1,23
2	i4	14	7,3	4,5	2,3	1,9	4,0	0,3	19,18	6209	1,54	72,93	1,26
3	i1	3	0,3	0,5	0,4	0,8	0,5	0,2	16,80	4266	3,53	85,00	0,93
3	i2	5	0,9	0,9	0,6	1	0,9	0,2	17,34	4391	0,66	80,80	1,36
3	i3	8	2,4	2,6	2,9	2,3	2,6	0,3	19,88	5709	2,36	76,25	1,24
3	i4	14	2,7	3,2	2,4	4	3,1	0,2	16,63	3919	2,30	82,14	1,17
4	i1	3	1,7	0,8	0,8	1,1	1,1	0,4	17,30	6265	0,00	72,33	1,50
4	i2	5	2,4	1,8	2	1,2	1,9	0,4	18,66	5169	0,00	74,60	1,18
4	i3	8	3,1	2,3	2,2	1,2	2,2	0,3	17,81	3346	3,40	83,25	0,88
4	i4	14	2,9	2,9	2,7	2,4	2,7	0,2	13,94	3785	0,48	82,00	1,03

Table S2. Numbers of BINs, OTUs, species, and accumulated BINs (BINsAcc) for each site and trapping interval of the local sampling. The numbers of BINs and BINsAcc are also given for subsets of high (taxa caught at 4 sites), medium (taxa caught at 2 or 3 sites), and low presence (taxa caught at 1 site).

						BIN subsets				BINAcc subsets		
Site	Interval	Days of trapping	BINs	OTUs	Species	Low	Medium	High	BINsAcc	Low	Medium	High
A	i1	3	130	232	78	25	52	53	130	25	52	53
A	i2	5	176	292	102	38	66	72	243	61	99	83
A	i3	8	191	323	107	37	80	74	332	94	141	97
A	i4	14	184	295	101	36	67	81	399	120	171	108
B	i1	3	75	120	39	16	18	41	75	16	18	41
B	i2	5	131	231	74	12	47	72	163	27	59	77
B	i3	8	233	403	116	49	95	89	311	73	134	104
B	i4	14	134	210	76	21	48	65	352	89	155	108
C	i1	3	100	171	53	17	33	50	100	17	33	50
C	i2	5	170	292	102	39	59	72	215	54	80	81
C	i3	8	235	383	122	62	85	88	343	106	135	102
C	i4	14	230	401	124	51	92	87	426	140	178	108
D	i1	3	91	159	53	11	34	46	91	11	34	46
D	i2	5	137	235	81	23	53	61	186	33	77	76
D	i3	8	178	287	97	41	70	67	281	65	121	95
D	i4	14	186	309	110	36	76	74	358	90	160	108

Table S3. Number of BINs for each site of the regional sampling. The numbers of BINs and BINsAcc are also given for subsets of high (taxa caught at 4 sites), medium (taxa caught at 2 or 3 sites), and low presence (taxa caught at 1 site).

		BIN subsets		
Site	BINs	Low	Medium	High
1	344	153	131	60
2	334	140	133	61
3	344	130	151	63
4	240	68	106	66
5	251	91	99	61
6	315	131	122	62
7	219	64	100	55
8	314	128	124	62
9	303	127	120	56
10	281	99	121	61
11	300	118	118	64
12	241	67	109	65
13	237	67	109	61
14	306	128	122	56
15	266	90	113	63
16	252	79	114	59
17	292	119	109	64
18	396	190	146	60
19	231	64	105	62
20	251	79	109	63
21	251	81	111	59
22	309	137	113	59
23	281	113	109	59
24	284	95	126	63
25	254	81	118	55
26	438	243	137	58
27	279	89	124	66
28	272	85	121	66
29	254	80	110	64
30	248	84	107	57
31	246	90	95	61
32	223	68	98	57

Supplementary Methods

S.1 DNA Metabarcoding

Species identification of organic material in the Malaise traps was performed using DNA metabarcoding following the protocol published in Hausmann et al. [1]. Each single sample was dried in a 60 °C oven for at least eight hours and subsequently homogenized in a FastPrep96 machine (MP Biomedicals) using sterile steal beads to generate a homogeneous mixture of animal material. Prior to DNA extraction, a subsample of each homogenate was transferred into sample vials and processed using the DNeasy 96 Blood & Tissue Kit (Qiagen) following the manufacturer's instructions. For amplification of the CO1-5P target region and preparation of the MiSeq libraries, a 2-step PCR was performed. First, a 313 bp long mini-barcode region was amplified by PCR, using forward and reverse HTS primers, equipped with complementary sites for the Illumina sequencing tails. In a subsequent PCR reaction, index primers with unique i5 and i7 inline tags and sequencing tails were used for amplification of indexed amplicons. Afterward, equimolar amplicon pools of 100 ng/µL each were created and size checked using preparative gel electrophoresis. The pooled DNA was purified using MagSi-NGSprep Plus beads (Steinbrenner Laborsysteme GmbH, Wiesenbach, Germany). A bioanalyzer (High Sensitivity DNA Kit, Agilent Technologies) was used for a final check of the bp distribution and concentration of the amplicons before the creation of the final library. High-throughput sequencing (HTS) was performed on an Illumina MiSeq using v2 (2*250 bp, 500 cycles, maximum of 20mio reads) chemistry (Illumina) aiming for 250k raw reads for each sample.

S.2 Bioinformatics

The bioinformatics processing of raw FASTQ files from Illumina was carried out using the *VSEARCH suite v.2.9.1* [2] and *Cutadapt v.1.18* [3]. Forward and reverse reads in each sample were merged using the *VSEARCH* program *fastq_mergepairs* with a minimum overlap of 10 bp, yielding approximately 313 bp long sequences. Forward and reverse primers were removed with *Cutadapt*, using the *discard_untrimmed* option to discard sequences for which primers were not reliably detected at ≥ 90 % identity. Quality filtering was done with the *fastq_filter* in *VSEARCH*, keeping sequences with zero expected errors (*fastq_maxee* 1). Sequences were dereplicated with *derep_fulllength*, first at the sample level and then concatenated into one FASTA file, which was subsequently dereplicated. Chimeric sequences were filtered out from the FASTA file using the *VSEARCH* program *uchime_denovo*. The remaining sequences were then clustered into OTUs at 97 % identity with *cluster_size*, a greedy centroid-based clustering program. OTUs were blasted against a custom Animalia database downloaded from BOLD in Q4 2021, including taxonomy and BIN information, by means of *Geneious v.10.2.5* (Biomatters, Auckland, New Zealand), and following methods described in Morinière et al. [4]. This local sequence database consists of the compiled data which are based on the DNA library with more than 23,000 barcoded German animal species assembled in two major DNA barcoding campaigns: "Barcoding Fauna Bavarica" (BFB, www.faunabavarica.de) and "German Barcode of Life" project (GBOL, www.bolgermany. de), with nearly 250,000 vouchers curated at the Zoological State Collection Munich, Germany (www.barcoding-zsm.de). Data releases have been published for all major arthropod groups (Coleoptera [5–8], Diptera [9], Ephemeroptera, Plecoptera, and Trichoptera [10], Heteroptera [11,12], Hymenoptera [13–15], Lepidoptera [16,17], Neuroptera [18], Orthoptera [19], Araneae and Opiliones [20], and Myriapoda [21,22]). The resulting CSV file which included the OTU ID, BOLD Process ID, BIN, Hit-%-ID value (percentage of overlap similarity (identical base pairs) of an OTU query sequence with its closest counterpart in the database), length of the top BLAST hit sequence, phylum, class, order, family, genus, and species information for each detected OTU was exported from *Geneious* and combined with the OTU table generated by the bioinformatic pipeline.

Supplementary References

1. Hausmann, A.; Segerer, A.H.; Greifenstein, T.; Knubben, J.; Morinière, J.; Bozicevic, V.; Doczkal, D.; Günter, A.; Ulrich, W.; Habel, J.C. Toward a standardized quantitative and qualitative insect monitoring scheme. *Ecol. Evol.* **2020**, *10*, 4009–4020, doi:10.1002/ece3.6166.
2. Rognes, T.; Flouri, T.; Nichols, B.; Quince, C.; Mahé, F. VSEARCH: a versatile open source tool for metagenomics. *PeerJ* **2016**, *4*, e2584, doi:10.7717/peerj.2584.
3. Martin, M. Cutadapt removes adapter sequences from high-throughput sequencing reads. *EMBnet j.* **2011**, *17*, 10, doi:10.14806/ej.17.1.200.
4. Morinière, J.; Cancian de Araujo, B.; Lam, A.W.; Hausmann, A.; Balke, M.; Schmidt, S.; Hendrich, L.; Doczkal, D.; Fartmann, B.; Arvidsson, S.; et al. Species Identification in Malaise Trap Samples by DNA Barcoding Based on NGS Technologies and a Scoring Matrix. *PLOS ONE* **2016**, *11*, e0155497, doi:10.1371/journal.pone.0155497.
5. Rulik, B.; Eberle, J.; Mark, L.; Thormann, J.; Jung, M.; Köhler, F.; Apfel, W.; Weigel, A.; Kopetz, A.; Köhler, J.; et al. Using taxonomic consistency with semi-automated data pre-processing for high quality DNA barcodes. *Methods Ecol Evol* **2017**, *8*, 1878–1887, doi:10.1111/2041-210X.12824.
6. Raupach, M.J.; Hannig, K.; Moriniére, J.; Hendrich, L. A DNA barcode library for ground beetles of Germany: the genus Amara Bonelli, 1810 (Insecta, Coleoptera, Carabidae). *Zookeys* **2018**, *759*, 57–80, doi:10.3897/zookeys.759.24129.

7. Raupach, M.J.; Hannig, K.; Morinière, J.; Hendrich, L. A DNA barcode library for ground beetles (Insecta, Coleoptera, Carabidae) of Germany: The genus Bembidion Latreille, 1802 and allied taxa. *Zookeys* **2016**, 592, 121–141, doi:10.3897/zookeys.592.8316.
8. Hendrich, L.; Morinière, J.; Haszprunar, G.; Hebert, P.D.N.; Hausmann, A.; Köhler, F.; Balke, M. A comprehensive DNA barcode database for Central European beetles with a focus on Germany: adding more than 3500 identified species to BOLD. *Molecular Ecology Resources* **2015**, *15*, 795–818, doi:10.1111/1755-0998.12354.
9. Morinière, J.; Balke, M.; Doczkal, D.; Geiger, M.F.; Hardulak, L.A.; Haszprunar, G.; Hausmann, A.; Hendrich, L.; Regalado, L.; Rulik, B.; et al. A DNA barcode library for 5,200 German flies and midges (Insecta: Diptera) and its implications for metabarcoding-based biomonitoring. *Molecular Ecology Resources* **2019**, *19*, 900–928, doi:10.1111/1755-0998.13022.
10. Morinière, J.; Hendrich, L.; Balke, M.; Beermann, A.J.; König, T.; Hess, M.; Koch, S.; Müller, R.; Leese, F.; Hebert, P.D.N.; et al. A DNA barcode library for Germany's mayflies, stoneflies and caddisflies (Ephemeroptera, Plecoptera and Trichoptera). *Molecular Ecology Resources* **2017**, *17*, 1293–1307, doi:10.1111/1755-0998.12683.
11. Raupach, M.J.; Hendrich, L.; Küchler, S.M.; Deister, F.; Morinière, J.; Gossner, M.M. Building-up of a DNA barcode library for true bugs (insecta: hemiptera: heteroptera) of Germany reveals taxonomic uncertainties and surprises. *PLOS ONE* **2014**, *9*, e106940, doi:10.1371/journal.pone.0106940.
12. Havemann, N.; Gossner, M.M.; Hendrich, L.; Morinière, J.; Niedringhaus, R.; Schäfer, P.; Raupach, M.J. From water striders to water bugs: the molecular diversity of aquatic Heteroptera (Gerromorpha, Nepomorpha) of Germany based on DNA barcodes. *PeerJ* **2018**, *6*, e4577, doi:10.7717/peerj.4577.
13. Schmidt, S.; Taeger, A.; Morinière, J.; Liston, A.; Blank, S.M.; Kramp, K.; Kraus, M.; Schmidt, O.; Heibo, E.; Prous, M.; et al. Identification of sawflies and horntails (Hymenoptera, 'Symphyta') through DNA barcodes: successes and caveats. *Molecular Ecology Resources* **2017**, *17*, 670–685, doi:10.1111/1755-0998.12614.
14. Schmidt, S.; Schmid-Egger, C.; Morinière, J.; Haszprunar, G.; Hebert, P.D.N. DNA barcoding largely supports 250 years of classical taxonomy: identifications for Central European bees (Hymenoptera, Apoidea partim). *Molecular Ecology Resources* **2015**, *15*, 985–1000, doi:10.1111/1755-0998.12363.
15. Schmid-Egger, C.; Straka, J.; Ljubomirov, T.; Blagoev, G.A.; Morinière, J.; Schmidt, S. DNA barcodes identify 99 per cent of apoid wasp species (Hymenoptera: Ampulicidae, Crabronidae, Sphecidae) from the Western Palearctic. *Molecular Ecology Resources* **2019**, *19*, 476–484, doi:10.1111/1755-0998.12963.
16. Hausmann, A.; Haszprunar, G.; Segerer, A.H.; Speidel, W.; Behounek, G.; Hebert, P.D.N. Now DNA-barcoded: the butterflies and larger moths of Germany. *Spixiana* **2011**, *34*, 47–58.
17. Hausmann, A.; Haszprunar, G.; Hebert, P.D.N. DNA barcoding the geometrid fauna of Bavaria (Lepidoptera): successes, surprises, and questions. *PLOS ONE* **2011**, *6*, e17134, doi:10.1371/journal.pone.0017134.
18. Morinière, J.; Hendrich, L.; Hausmann, A.; Hebert, P.; Haszprunar, G.; Gruppe, A. Barcoding Fauna Bavarica: 78% of the Neuropterida fauna barcoded! *PLOS ONE* **2014**, *9*, e109719, doi:10.1371/journal.pone.0109719.
19. Hawlitschek, O.; Morinière, J.; Lehmann, G.U.C.; Lehmann, A.W.; Kropf, M.; Dunz, A.; Glaw, F.; Detcharoen, M.; Schmidt, S.; Hausmann, A.; et al. DNA barcoding of crickets, katydids and grasshoppers (Orthoptera) from Central Europe with focus on Austria, Germany and Switzerland. *Molecular Ecology Resources* **2017**, *17*, 1037–1053, doi:10.1111/1755-0998.12638.
20. Astrin, J.J.; Höfer, H.; Spelda, J.; Holstein, J.; Bayer, S.; Hendrich, L.; Huber, B.A.; Kielhorn, K.-H.; Krammer, H.-J.; Lemke, M.; et al. Towards a DNA Barcode Reference Database for Spiders and Harvestmen of Germany. *PLOS ONE* **2016**, *11*, e0162624, doi:10.1371/journal.pone.0162624.
21. Wesener, T.; Voigtländer, K.; Decker, P.; Oeyen, J.P.; Spelda, J.; Lindner, N. First results of the German Barcode of Life (GBOL) - Myriapoda project: Cryptic lineages in German Stenotaenialinearis (Koch, 1835) (Chilopoda, Geophilomorpha). *Zookeys* **2015**, *510*, 15–29, doi:10.3897/zookeys.510.8852.
22. Spelda, J.; Reip, H.S.; Oliveira-Biener, U.; Melzer, R.R. Barcoding Fauna Bavarica: Myriapoda - a contribution to DNA sequence-based identifications of centipedes and millipedes (Chilopoda, Diplopoda). *Zookeys* **2011**, *156*, 123–139, doi:10.3897/zookeys.156.2176.
